# Hierarchically Multivalent Peptide–Nanoparticle Architectures: A Systematic Approach to Engineer Surface Adhesion

**DOI:** 10.1002/advs.202103098

**Published:** 2021-12-11

**Authors:** Woo‐jin Jeong, Jiyoon Bu, Roya Jafari, Pavel Rehak, Luke J. Kubiatowicz, Adam J. Drelich, Randall H. Owen, Ashita Nair, Piper A. Rawding, Michael J. Poellmann, Caroline M. Hopkins, Petr Král, Seungpyo Hong

**Affiliations:** ^1^ Pharmaceutical Sciences Division and Wisconsin Center for NanoBioSystems (WisCNano) School of Pharmacy University of Wisconsin‐Madison 777 Highland Ave Madison WI 53705 USA; ^2^ Department of Biological Sciences and Bioengineering Inha University 100 Inha‐ro, Michuhol‐gu Incheon 22212 Republic of Korea; ^3^ Department of Chemistry University of Illinois at Chicago 845 W Taylor St Chicago IL 60607 USA; ^4^ Departments of Physics, Pharmaceutical Sciences and Chemical Engineering University of Illinois at Chicago 845 W Taylor St Chicago IL 60607 USA; ^5^ Department of Biomedical Engineering The University of Wisconsin‐Madison 1550 Engineering Dr. Madison WI 53705 USA; ^6^ Yonsei Frontier Lab Department of Pharmacy Yonsei University 50 Yonsei‐ro, Seodaemun‐gu Seoul 03722 Republic of Korea

**Keywords:** binding avidity, dendrimer–peptide conjugate, hierarchically multivalent architectures, multivalent binding, peptide engineering

## Abstract

The multivalent binding effect has been the subject of extensive studies to modulate adhesion behaviors of various biological and engineered systems. However, precise control over the strong avidity‐based binding remains a significant challenge. Here, a set of engineering strategies are developed and tested to systematically enhance the multivalent binding of peptides in a stepwise manner. Poly(amidoamine) (PAMAM) dendrimers are employed to increase local peptide densities on a substrate, resulting in hierarchically multivalent architectures (HMAs) that display multivalent dendrimer–peptide conjugates (DPCs) with various configurations. To control binding behaviors, effects of the three major components of the HMAs are investigated: i) poly(ethylene glycol) (PEG) linkers as spacers between conjugated peptides; ii) multiple peptides on the DPCs; and iii) various surface arrangements of HMAs (i.e., a mixture of DPCs each containing different peptides vs DPCs cofunctionalized with multiple peptides). The optimized HMA configuration enables significantly enhanced target cell binding with high selectivity compared to the control surfaces directly conjugated with peptides. The engineering approaches presented herein can be applied individually or in combination, providing guidelines for the effective utilization of biomolecular multivalent interactions using DPC‐based HMAs.

## Graphical abstract

This study presents a novel surface platform that utilizes hierarchically controlled multivalent binding of peptides. Using dendrimers, peptides, and linkers as building blocks, the role of each component and the effect of surface presentation/arrangement of different peptides on surface binding are investigated. The results presented herein provide guidelines for the effective use of biomolecular multivalent interactions for various biomedical applications.

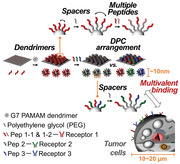

## 1 Introduction

The majority of biological events (e.g., protein folding, signal transduction, material transport, and viral infection) occur through binding/unbinding processes among biological substances.^[^ ^1^ ^]^ Such binding events often utilize co‐operative interactions, also known as multivalent interactions, to exponentially enhance binding strength via avidity when needed.^[^ ^2^ ^]^ As a result, the naturally occurring multivalent binding effect enables otherwise relatively weak noncovalent forces to become strong enough to control various biological consequences. Presenting multiple copies of ligands, multivalent biomaterials typically communicate with their counterparts through combinations of various binding elements, which improves the kinetic and thermodynamic aspects of the interactions.^[^ ^3^ ^]^ The spatial arrangement of ligands is another major factor that affects multivalent binding.^[^ ^4^ ^]^ For instance, the detection of pathogens by the innate immune system is achieved by the multivalent interaction between T cell receptors and antigens present on a pathogen, which is highly affected by the density and array of antigenic fragments.^[^ ^5^ ^]^ To regulate such biological phenomena, it is therefore important to fully understand fundamental principles of multivalent interactions and to devise effective engineering strategies to control them, which would be useful for a wide range of biomedical research.

Given their protein‐like functions, low‐cost, and modular nature, peptides have been extensively studied to facilitate specific biological interactions.^[^ ^6^, ^7^ ^]^ Peptides can be synthesized with fine‐tuned amino acid sequences and nanoscale topologies, allowing them to have controlled orientation and folding structure when conjugated to a substrate and, in turn, effectively bind to target molecules.^[^ ^7^, ^8^, ^9^ ^]^ However, implementation of peptides to various biomedical applications has been hindered primarily due to their limited binding strength that is typically inferior to their whole antibody counterparts.^[^ ^10^ ^]^ This issue could be addressed via the incorporation of otherwise weakly binding peptides to nanoparticles that are engineered to mediate strong multivalent binding. Among various nanoparticles, poly(amidoamine) (PAMAM) dendrimers serve as an excellent platform to mediate a biomolecular multivalent binding effect owing to their hyperbranched, chemically well‐defined structure with a high degree of deformability.^[^ ^11^, ^12^ ^]^ Using generation 7 (G7) PAMAM dendrimers conjugated with peptides, we have recently demonstrated that the dendrimer–peptide conjugates (DPCs) exhibit drastically enhanced binding avidity by reducing the dissociation kinetics (up to 5 orders of magnitude), compared to free peptides.^[^ ^8^ ^]^ However, precise control over the strong adhesion of peptides using the nanoparticle‐mediated multivalent binding effect remains elusive.

**Figure 1:**
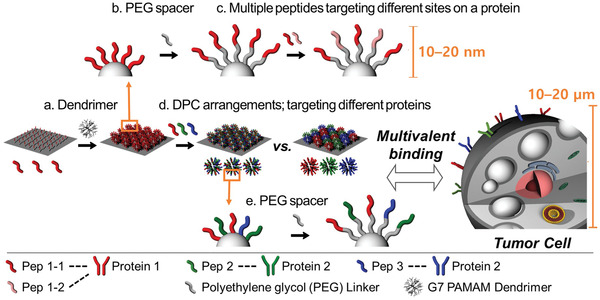
Schematic illustration of the stepwise engineering strategies applied to control the multivalent binding behaviors of various peptides with G7 PAMAM dendrimers: a) hyperbranched PAMAM dendrimers were employed to increase local peptide density; b) PEG spacers were introduced to spatially segregate the peptides from the dendrimer and to increase the conformational freedom of the peptides; c) multiple peptide sequences that bind to different sites within a single protein were coimmobilized to the dendrimer‐PEG surfaces to enhance the target binding; d) two different HMA surfaces, a mixture of DPCs each containing different peptides (HoMA) and DPCs cofunctionalized with multiple peptides (HeMA), were tested to investigate the effect of the peptide arrangement on their hetero‐multivalent binding behaviors; e) PEG linkers were introduced to hetero‐HMAs to explore whether the use of spacer molecules on HMA synergize the multivalent binding effect.

In this study, we have devised systematic engineering strategies to maximize the multivalent binding effect of peptides mediated by dendrimers in a controlled manner (**Figure** **1**). A series of hierarchically multivalent architectures (HMAs), i.e., multivariant arrays of the multivalent DPCs, were constructed using DPCs. The binding behaviors of HMAs were observed upon the interaction of the surfaces with cells in vitro. We first optimized the surface configuration of individual DPCs by introducing poly(ethylene glycol) (PEG) spacers to spatially segregate the peptides from the dendrimer surface and to increase interpeptide distance for conformational freedom. Various molecular weight PEG spacers were tested to determine the most effective configuration that maximizes the number of accessible amino acids. Second, multiple peptide sequences that bind to different sites within a single protein were immobilized to the dendrimer–PEG surfaces, expecting that their co‐operative interactions would enhance the overall binding strengths of the functionalized surfaces. Third, we also constructed heterogeneous HMAs (hetero‐HMAs) that display various peptides interacting with different types of proteins, which could increase the total number of peptide–protein binding pairs between the functionalized surfaces and cells. Two distinct hetero‐HMAs (a heterogeneous mixture of homogeneous DPCs, HoMA; a homogeneous mixture of heterogeneous DPCs, HeMA) were prepared to investigate the effect of different peptide arrangements on hetero‐multivalent binding behaviors. Additionally, we explored whether the use of spacer molecules could improve the multivalent binding behaviors of the hetero‐HMAs. These engineering strategies tested herein could be utilized individually or in combination, which would potentially allow for the development of multivalent peptide‐based nanostructures that are fine‐tuned for an optimal configuration to achieve desirable binding avidity tailored to various applications.

## 2 Results and Discussion

This study tested the effects of: i) surface‐immobilized dendrimers on the sensitivity and specificity of cell binding; ii) PEG linkers as spacers; and iii) the surface configuration of dendrimers functionalized with individual or multiple peptides. All experiments were performed on substrates functionalized with single or multiple types of peptides that are immobilized through various functional layers, including dendrimers and PEGs, to realize precisely engineered surface presentations of the peptides of interest. G7 PAMAM dendrimers were exploited as a representative multivalent scaffold since G7 is reported to be the highest generation dendrimer with well‐defined molecular architecture.^[^ ^13^ ^]^ It should be noted that in vitro tumor cell lines were employed throughout this study to assess the avidity and selectivity of the HMAs to target proteins, as the cells are microscale particles (10–20 µm) decorated with many different proteins that can accommodate a number of molecular interactions simultaneously.^[^ ^14^ ^]^

### 2.1 Surface Configuration Engineering of Individual DPCs

**Figure 2:**
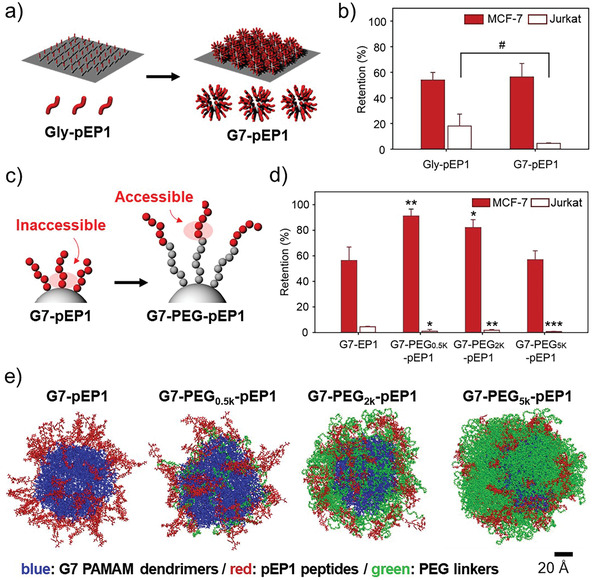
Surface engineering of individual DPCs: a) Introduction of G7 PAMAM dendrimers as a building block of HMA formation. b) % retention of surface‐bound MCF‐7 (EpCAM^high^; red) and Jurkat (EpCAM^negative^; white) cells on Gly‐pEP1 versus G7‐pEP1 upon washing with a high shear flow. c) Schematic illustration of anticipatedly enhanced accessibility of pEP1 with the introduction of PEG spacers on the dendrimer surface. d) Retention of surface‐bound MCF‐7 and Jurkat cells on the HMA surfaces with various molecular weight PEG spacers upon washing. e) Molecular dynamics (MD) modeling of G7‐PEG‐pEP1 configurations with various PEG outer layers after 40 ns of simulation time. All washing steps for cell retention assays were performed at a flow rate of 50 µL min^−1^, which corresponds to a shear stress of 0.36 dyne cm^−2^. Significance levels are indicated as ^#^ *p* < 0.10, ^*^ *p* < 0.05, ^**^ *p* < 0.01, and ^***^ *p* < 0.001, which are analyzed using Student's *t*‐test.

We first examined the effect of dendrimers using epithelial cell adhesion molecule (EpCAM)‐binding peptides (pEP1; Figures S1–S3, Supporting Information).^[^ ^15^ ^]^ EpCAM was chosen as the first target to investigate since it is commonly expressed by a wide variety of human epithelial carcinoma.^[^ ^16^ ^]^ The pEP1 peptides were immobilized onto substrates functionalized with either short glycine linkers (Gly‐pEP1) or G7 PAMAM dendrimers (G7‐pEP1) (**Figure** **2**a). The binding strengths between the functionalized surfaces and cells were assessed using a cell retention assay, as described in our earlier publications.^[^ ^12^ ^]^ Briefly, peptide‐immobilized surfaces were assembled into a flow chamber (Figure S4, Supporting Information), in which cells were infused and incubated for 30 min, followed by a 20 min wash using phosphate‐buffered saline (PBS) at a flow rate of 50 µL min^−1^ that corresponds to a shear stress of 0.36 dyne cm^−2^). The retention efficiency (*E*_Ret_) was determined as a ratio of the cells remained on a surface after washing to the cells that were initially bound to the same surface. More details can be found in the Supporting Information. The dendrimer‐coated surfaces allowed for a slightly greater amount of pEP1 to be surface‐immobilized than the glycine linkers (Figure S5, Supporting Information; ≈1.1‐fold), although this was not enough to lead to higher retention of EpCAM^High^ MCF‐7 cells (Figure 2b; 56.4 ± 10.5% vs 53.9 ± 5.9%; *p* = 0.744). In contrast, noncancerous EpCAM^Negative^ Jurkat cells exhibited lower *E*_Ret_ on G7‐pEP1 than Gly‐pEP1 (4.5 ± 0.4% vs 18.1 ± 9.3%; *p* = 0.065), indicating that the dendrimer surface (HMA) blocks nonspecific binding more efficiently than the glycine surface (non‐HMA). Despite the marginal increase in sensitivity, this result indicates that G7‐pEP1 exhibits improved selectivity toward MCF‐7 cells, justifying our use of HMA (surfaces with dendrimers) for subsequent experiments.

To enhance tumor cell binding to the HMA, PEG spacers were employed, as depicted in Figure 2c. When peptides are directly conjugated to a dendrimer, the amino acid residues adjacent to the surface are typically inaccessible to bind to target proteins due to steric hindrance,^[^ ^17^ ^]^ which often reduces binding affinity. The PEG spacers were thus used to improve accessibility, which increased the possibility of the amino acid residues to participate in the peptide–protein interactions (Figure 2c). The *E*_Ret_ of MCF‐7 cells (Figure 2d) was significantly increased when PEG_0.5k_ chains were placed between dendrimers and peptides (91.1 ± 5.4%). Interestingly, further increases in the molecular weight of PEG resulted in decreased cell binding. The *E*_Ret_ decreased to ≈82% for PEG_2k_ (*p* = 0.129 compared to PEG_0.5k_) and ≈57% for PEG_5K_ (*p* = 0.003) (Table S1, Supporting Information). This observation is consistent with the results presented in our previous publication where peripheral amine groups of PEG_2K_ conjugated to a dendron‐micelle exhibited a significantly lower level of interactions with tumor cells than those on PEG_0.6K_.^[^ ^18^ ^]^ Furthermore, there have been a number of reports suggesting that the back‐folding of the long polymer chains (mushroom structure) conceal the reactive amines into the PEG layer, whereas shorter PEG molecules maintain a less‐folded “brush structure,” allowing these amine groups to be exposed (or “accessible”) on the surface.^[^ ^19^ ^]^ Considering that the contour length of a PEG_5K_ chain (31.8 nm)^[^ ^20^ ^]^ is significantly longer than that of pEP1 (3.3 nm; Figure S6, Supporting Information), the reduced *E*_Ret_ of G7‐PEG_5k_‐pEP1 is likely attributed to the conformational difference of the polymers depending on the PEG length.

The molecular dynamics (MD) modeling further supports this argument.^[^ ^34^ ^]^ That is, more of the pEP1 peptides (red) were exposed on the surface of DPCs without PEG or with PEG_0.5K_ (Figure 2e). In contrast, G7‐PEG_2k_‐pEP1 displayed the peptides embedded into the PEG outer layers, which was further augmented with G7‐PEG_5k_‐pEP1. The “inaccessibility” of the peptides within the longer PEG chains thus diminishes the chances of peptides to interact with target proteins, likely resulted in the weak cell adhesion of the HMAs observed. The MD simulations also demonstrated that the PEG linkers increased the conformational freedom of the peptides (reachability), whereas a strong rigidity of G7‐pEP1 was only maintained when the short PEG_0.5K_ linkers were used (Videos S1–S4, Supporting Information). With the longer PEG chains, DPCs lost their sufficient rigidity that is required to establish multivalency.^[^ ^18^, ^21^ ^]^ In addition, the longer PEG chains increased the distance between the neighboring peptides, which in turn reduced the peptide concentration on the dendrimer surface and decreased the multivalent binding effect.^[^ ^22^ ^]^

We also found that nonspecific binding of Jurkat cells was significantly reduced in the presence of PEG linkers, regardless of their chain length (*E*_Ret_ < 1.8%; *p* < 0.012 vs HMAs without PEG). This appears to be directly related to the threshold molecular weight of PEG to function as a nonfouling polymer. When the thickness of the polymer layer is larger than the size of a protein, the equilibrium amount of protein that can adsorb on the polymer surface is independent of its thickness.^[^ ^23^ ^]^ Considering that the length of G7‐PEG_0.5k_ (diameter of ≈8.1 nm for G7 and contour length of >3.1 nm for PEG_0.5k_) exceeds the size of the majority of human proteins, the length of PEG_0.5k_ should be sufficient to shield the nonspecific protein adsorption and prevent the nonselective cell binding.

**Figure 3:**
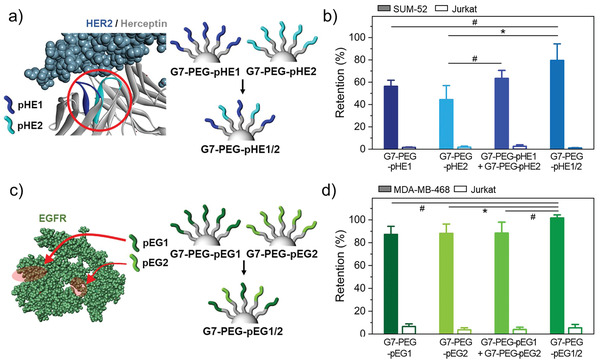
Dendrimer‐PEG_0.5k_ surfaces coimmobilized with multiple peptide sequences that bind to different sites within a single protein: a) Schematic illustration of G7‐PEG surfaces immobilized with two different peptide sequences that bind to different sites of HER2 protein, pHE1 and pHE2. b) Retention of surface‐bound SUM‐52 and Jurkat cells on HMA surfaces consisting of G7‐PEG_0.5k_‐pHE1, G7‐PEG_0.5k_‐pHE2, a mixture of the two DPCs, or G7‐PEG_0.5k_‐pHE1/2 upon washing. c) Schematic illustration of G7‐PEG_0.5k_ surfaces immobilized with two different peptide sequences that bind to different sites of EGFR protein, pEG1 and pEG2. d) Retention of surface‐bound MDA‐MB‐468 and Jurkat cells on HMA surfaces consisting of G7‐PEG_0.5k_‐pEG1, G7‐PEG_0.5k_‐pEG2, a mixture of the two DPCs, or G7‐PEG_0.5k_‐pEG1/2 upon washing. All washing steps for cell retention assays were performed at a flow rate of 50 µL min^−1^, which corresponds to a shear stress of 0.36 dyne cm^−2^. Significance levels are indicated as ^#^ *p* < 0.10, ^*^ *p* < 0.05, ^**^ *p* < 0.01, and ^***^ *p* < 0.001, which are analyzed using Student's *t*‐test.

Next, multiple peptide sequences that bind to different sites within a single protein were coimmobilized to a PEGylated DPC to further enhance the binding avidity of HMAs. As the pEP1 peptides on G7‐PEG_0.5k_ already achieved over 90% *E*_Ret_ for MCF‐7 cells, we utilized pairs of peptide sequences targeting other cancer‐specific proteins, human epidermal growth factor receptor 2 (HER2) and epidermal growth factor receptor (EGFR). Note that the HER2‐binding peptides (pHE1 and pHE2)^[^ ^24^ ^]^ and EGFR‐binding peptides (pEG1 and pEG2)^[^ ^25^ ^]^ were synthesized via structure‐based molecular design and library screening methods, respectively, which are the two major chemical techniques for the identification of peptide sequences (Figures S1–S3, Supporting Information). The combination of the two Herceptin‐derived peptide sequences (HER2‐binding peptides) on HMAs (G7‐PEG_0.5k_‐pHE1/2) enhanced the cell binding, exhibiting higher *E*_Ret_ for HER2^High^ SUM‐52 cells (79.5 ± 14.8%) than HMAs with either of the two peptides (G7‐PEG_0.5k_‐pHE1: 56.3 ± 5.5% and G7‐PEG_0.5k_‐pHE2: 44.4 ± 12.6% with *p* values of 0.064 and 0.035, respectively) (**Figure** **3**a,b). This configuration was also more effective than combining the two DPCs each immobilized with a different type of HER2‐targeting peptides (G7‐PEG_0.5k_‐pHE1 + G7‐PEG_0.5k_‐pHE2), which had an *E*_Ret_ of 63.4 ± 7.1%. A similar trend was also observed for the EGFR‐binding peptides. The coimmobilization of pEG1/pEG2 on the G7‐PEG_0.5k_ (G7‐PEG_0.5k_‐pEG1/2) enhanced the retention of EGFR^High^ MDA‐MB‐468 cells by 1.17‐fold (*p* = 0.030) and 1.15‐fold (*p* = 0.053) compared to pEG1 and pEG2, respectively. In contrast, the physical mixture of the two DPCs exhibited a similar level of *E*_Ret_ to the single type of DPCs (Figure 3c,d; Figure S7, Supporting Information). These findings suggest that the intermolecular distance between the different peptide sequences coimmobilized on the PEGylated dendrimer is close enough to form a strong multivalent interaction with the target receptor. However, the multivalent binding effect was less pronounced from the peptide sequences immobilized on different dendrimers. A similar phenomenon has been reported in a recent study where the silicon nanoparticles coimmobilized with two integrin‐binding peptide epitopes exhibited stronger cell‐binding compared to a mixture of the nanoparticles each immobilized with different peptides.^[^ ^26^ ^]^ Altogether, these results reveal that the coimmobilization of various peptide sequences, which can target different parts of a protein on a dendrimer increases the binding avidity of DPCs and thus enhances the cancer cell targeting.

### 2.2 Enhanced Multivalent Binding Using Hetero‐HMAs

**Figure 4:**
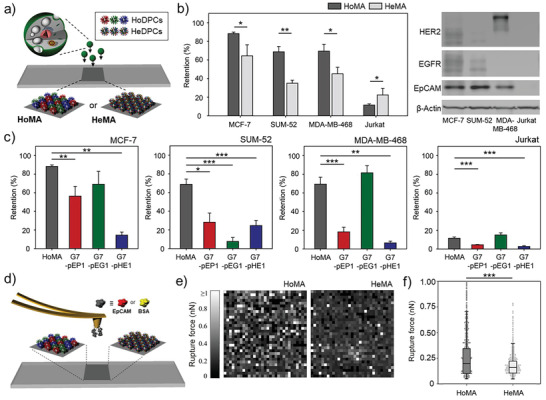
Two distinct hetero‐HMAs, HoMA (a heterogeneous mixture of homogeneous DPCs) and HeMA (a homogeneous mixture of heterogeneous DPCs), which display various peptides interacting with different types of proteins: a) A schematic illustration of HoMA and HeMA surfaces. b) The retention of surface‐bound MCF‐7, SUM‐52, MDA‐MB‐468, and Jurkat cells on HoMA and HeMA surfaces (left). Western blot analysis of EpCAM, HER2, and EGFR expression levels on the cell lines used in this study (right). c) The retention of surface‐bound MCF‐7, SUM‐52, MDA‐MB‐468, and Jurkat cells on HoMA compared to HMAs functionalized with a single type of peptides. d) A schematic illustration of atomic force microscopy (AFM) force mapping on HoMA and HeMA. e) AFM force mapping analysis using EpCAM‐immobilized probes to demonstrate the binding avidity of HoMA and HeMA against EpCAM protein. A 32 × 32 grid of retraction curves were obtained in the desired area of 10 × 10 µm^2^. f) Rupture forces based on AFM adhesion forces measured using EpCAM‐immobilized probes on HoMA and HeMA. Significance levels are indicated as ^*^ *p* < 0.05, ^**^ *p* < 0.01, and ^***^ *p* < 0.001, which are analyzed using Student's *t* test (cell retention) or the Mann–Whitney *U* test (AFM analysis).

Next, we constructed hetero‐HMAs with various peptides that target different proteins to further increase the binding interactions between HMAs and tumors cells, since cell membranes are loaded with many different types of proteins that perform a variety of functions. Two distinct hetero‐HMAs (**Figure** **4**a; Figures S8–S11, Supporting Information) were prepared: a heterogeneous mixture of homogeneous DPCs (HoMA; a mixture of G7‐pEP1, G7‐pEG1, and G7‐pHE1) and a homogeneous mixture of heterogeneous DPCs (HeMA; pEP1, pEG1, and pHE1 coimmobilized on a DPC at 1:1:1 ratio). Note that the ratio between pEP1, pEG1, and pHE1 on HeMA surface was 1.00:1.11:1.25, which is close to the equal distribution of the three peptides.

Using a flow chamber assay, significant differences in cell retention between the two hetero‐HMAs were quantitatively measured (Figure 4b; Figure S12, Supporting Information). Interestingly, HoMA exhibited significantly stronger binding to all three cancer cell lines used in this study than HeMA did (*p* < 0.050). This was exactly the opposite to the results obtained in the previous section, where the peptides targeting the different sites within a single protein exhibited stronger target binding when different peptides were coimmobilized on the same dendrimers. HoMA also outperformed HMAs consisting of a single type of DPCs (G7‐pEP1, G7‐pEG1, or G7‐pHE1) in capturing MCF‐7 and SUM‐52 cells (Figure 4c). MDA‐MB‐468 cells that overexpress EGFR with a low‐to‐weak degree of EpCAM and HER2 expressions were the only exception. However, the slightly higher *E*_Ret_ of the G7‐pEG1 surface than that of HoMA was still statistically insignificant (*p* = 0.120).

To further investigate the different binding behaviors of the HoMA and HeMA surfaces, atomic force microscopy (AFM) adhesion force mapping was performed as it could resolve the nano‐Newton‐scale binding forces generated from the two hetero‐HMAs (Figure 4d).^[^ ^27^ ^]^ As demonstrated in Figure 4e,f and Figure S13 in the Supporting Information, HoMA exhibited a stronger adhesion to EpCAM than HeMA with a wider force variation. Specifically, the median rupture force (interquartile range (IQR)) with the probe‐immobilized EpCAM was 0.20 (0.10–0.34) nN and 0.16 (0.11–0.21) nN for HoMA and HeMA, respectively (*p* < 0.001). The different binding behavior of the two hetero‐HMAs is attributed to their difference in peptide distribution. HoMA, which consists of the three homogeneous DPCs randomly distributed on a surface, may likely form clusters of DPCs having the same type of peptides (hierarchical cluster of peptides), whereas the three peptides are more uniformly distributed across the surface for HeMA. The distinct distributions of the peptides on HoMA and HeMA were analyzed using a computer‐aided simulation. Prior to the analysis, we confirmed that ≈90% of the surfaces of both hetero‐HMAs are occupied with DPCs, which was observed from AFM surface topography (Figure S14, Supporting Information). Based on this result, the HoMA and HeMA surfaces were modeled by assuming that each DPC occupies 1 × 1 pixels^2^. The HoMA surface was constructed by randomly filling 90% of 400 × 400 pixels^2^ with an RGB value of either (1,0,0) for G7‐pEP1, (0,1,0) for G7‐pEG1, (0,0,1) for G7‐pHE1. Meanwhile, the HeMA surface was modeled by filling 90% of the pixels with an RGB value of (0.33,0.33,0.33), which represents the DPCs coimmobilized with three different peptides (HeDPC) at a 1:1:1 ratio. As demonstrated in Figure S15 in the Supporting Information, HoMA consisted of red, blue, and green pixels, while HeMA was covered with gray pixels. For any type of peptide, the average peptide density on the randomly chosen local region (10 × 10 pixels^2^) was 29.7% for both HMAs. However, HoMA showed a significantly wider variation in local peptide density than HeMA, with an IQR of 5.8% versus 1.1%. The wider variation in local peptide density was indicative of HoMA having clusters of the same type of peptides across the surface. This “hierarchical cluster of peptides” on HoMA is conceived to form a strong multivalent binding with cellular proteins that circulate the plasma membrane.^[^ ^28^ ^]^

It has been also reported that when cellular proteins find their counter ligands, the proteins tend to recruit near the ligands and form adhesion complexes.^[^ ^28^ ^]^ This multivalent interaction between proteins–ligands is known to strengthen the adhesion between the cell and its counter object.^[^ ^29^ ^]^ Likewise, HoMA would likely form a stronger coupling with the cellular proteins than HeMA. For example, EGFR^High^ MDA‐MB‐468 cells may form a strong binding on a region having clusters of pEG1 peptides on HoMA, whereas the cells form a weaker bond on HeMA as pEG1 peptides are evenly distributed across the surface.

**Figure 5:**
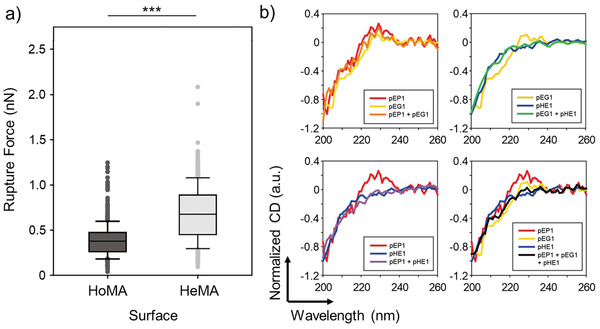
Nonspecific binding on the two hetero‐HMAs: a) Rupture forces between BSA‐immobilized probes on HoMA and HeMA, as quantified using AFM force measurements. b) CD spectra of the peptides (individual or in mixture) in PBS. The strong negative bands at ≈200 nm and the disappearance of the peak at 220–230 nm are indicative of structural changes due to the interpeptide interaction. Significance levels are indicated as ^*^ *p* < 0.05, ^**^ *p* < 0.01, and ^***^ *p* < 0.001, which are analyzed using the Mann–Whitney *U* test.

Next, we wanted to elucidate the mechanism behind the reduced nonspecific binding of Jurkat cells on HoMA, compared to HeMA (Figure 4b). The AFM force measurements were again performed under the same conditions but this time using BSA‐immobilized probes to quantitatively measure the nonspecific interactions occurring on the two hetero‐HMAs (**Figure** **5**a). We found that adhesion with BSA was significantly weaker on HoMA than that on HeMA (0.43 ± 0.02 nN vs 0.79 ± 0.03 nN; *p* < 0.001), which was in a good agreement with the measured values from our previous cell retention experiments using Jurkat cells (11.5 ± 1.3% vs 17.0 ± 2.5%; *p* = 0.028; Figure 4b). The strong nonspecific interaction on HeMA is likely due to its higher complexity/irregularity in amino acid arrangements, as a result of heterogeneous peptides coimmobilized on the same dendrimer (Figure S16, Supporting Information). Furthermore, the interpeptide interactions resulting from various attractive and repulsive forces between the neighboring peptides on a dendrimer may have altered the folding structure of the peptides on HeMA and therefore increased the nonspecific interactions. Circular dichroism (CD) spectroscopy was employed to verify the secondary structure changes due to these interpeptide interactions. The CD results revealed that when mixed with pHE1 in PBS, pEP1 and pEG1 peptides were unstructured as random coils which were demonstrated by the strong negative CD band at ≈200 nm and disappearance of CD peak at 220–230 nm,^[^ ^30^ ^]^ implying that pHE1 interacts with the other two peptides and altered their secondary structures (Figure 5b). These changes in the secondary structures may impact the binding behaviors of the peptides, increasing the nonspecific binding of Jurkat cells and reducing the capture of cancer cells.

**Figure 6:**
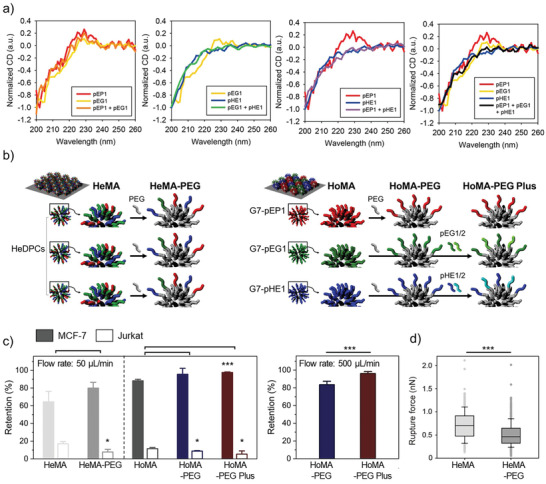
A strategy utilizing PEG linkers to enhance binding avidity and selectivity of hetero‐HMAs: a) A schematic illustration of the incorporation of the PEG linkers to increase interpeptide distances of HMAs. b) The retention of surface‐bound MCF‐7 and Jurkat cells on the HMAs and PEGylated HMAs upon washing at a flow rate of 50 µL min^−1^, which corresponds to a maximum shear stress of 0.36 dyne cm^−2^. c) The retention of surface‐bound MCF‐7 cells upon washing at a flow rate of 500 µL min^−1^, which corresponds to a shear stress of 3.6 dyne cm^−2^. d) AFM adhesion force measurements on HeMA versus PEGylated HeMA using a BSA‐immobilized probe. Significance levels are indicated as ^*^ *p* < 0.05, ^**^ *p* < 0.01, and ^***^ *p* < 0.001, which are analyzed using Student's *t*‐test (cell retention) or the Mann–Whitney *U* test (AFM analysis).

We next investigated the synergistic effect of utilizing PEG linkers on the hetero‐HMAs, by implementing PEG_0.5K_ spacers on HeMA and HoMA (**Figure** **6**a). The surface immobilization of dendrimers, PEG linkers, and peptides was confirmed using X‐ray photoelectron spectroscopy (XPS), contact angle measurement system, and AFM (Figure S17, Supporting Information). The PEGylated HeMA demonstrated 1.24‐fold (*p* = 0.118), 1.52‐fold (*p* = 0.006), and 1.46‐fold (*p* = 0.057) increased *E*_Ret_ for MCF‐7, SUM‐52, and MDA‐MB‐468 cells, respectively, compared to HeMA without PEG (Figure 6b; Figure S18, Supporting Information). Likewise, the PEGylated HoMA also exhibited 1.08‐fold increased *E*_Ret_ for MCF‐7, although the results were statistically less significant (*p* = 0.144). The binding avidity of the HMAs was further enhanced by coimmobilizing multiple peptide sequences that bind to different sites within a single protein on PEGylated dendrimers, and combining these DPCs each targeting different proteins (the mixture of G7‐PEG‐pEP1, G7‐PEG‐pHE1/2, and G7‐PEG‐pEG1/2; PEGylated HoMA‐plus). The PEGylated HoMA‐plus demonstrated the highest cell binding with *E*_Ret_ of 97.6 ± 0.6% for MCF‐7 cells. The enhanced cell binding of the PEGylated HoMA‐plus was more pronounced at a stronger shear condition (flow rate of 500 µL min^−1^), as the PEGylated HoMA‐plus exhibited 1.15‐fold stronger binding to MCF‐7 cells than the PEGylated HoMA (*p* = 0.008) (Figure 6c). The utilization of PEG linkers was also advantageous for reducing the nonspecific binding of Jurkat cells on both the HeMA and HoMA surfaces (Figure 6b). AFM force spectroscopy confirmed the antifouling effect of PEG spacers as the PEGylated HeMA exhibited ≈26% less adhesion with BSA‐immobilized probes than the surface without PEG linker (*p* < 0.001) (Figure 6d). All these findings collectively indicate that the PEG spacers increase the interpeptide distance, which furthers improves the specific adhesion of the hetero‐HMAs to their target proteins.

To verify whether these strategies can also be applied to other types of dendrimers having different ligand configurations, we investigated the spacing effect of PEG linkers and surface arrangement of different peptides on G4 PAMAM dendrimers. Note that G4 PAMAM dendrimers exhibit the lowest number of functional groups among the 3D rigid structured PAMAM dendrimers (≥G4), which have 35–50% functional groups per surface area compared to G7.^[^ ^13^, ^31^ ^]^ Although the spacer effect was not strongly observed as G7, G4 also had the highest *E*_Ret_ when PEG_0.5k_ chains were placed between dendrimers and peptides (Figure S19a, Supporting Information). This observation is also consistent with the previous study, which demonstrated that the amine groups of PEG_0.6k_‐conjugated G4 PAMAM dendron micelles induce stronger cellular interaction than those of PEG_2k_‐conjugated dendrimers.^[^ ^18^ ^]^ Likewise, when comparing HoMA versus HeMA configurations, the G4‐HoMA‐PEG surface adhered to the target cells more effectively than G4‐HeMA‐PEG (75.9 ± 6.9% vs 60.0 ± 14.6%; *p* = 0.164) (Figure S19b, Supporting Information). For other types or generations of dendrimers, these effects may vary depending on the dendrimer's size, flexibility, surface charge, the distance between the ligand, and the number of functional groups. However, the strategies provided herein give an idea and design cues for different dendrimers or other hyperbranched polymers to be employed in various biomedical applications with significantly enhanced binding affinity and selectivity.

## 3 Conclusion

This study presents novel engineering strategies that enable to control the binding behaviors of DPCs. Various combinations of the three primary components, i.e., dendrimers, PEGs, and peptides, were investigated to elucidate the roles of each component playing in enhancing cell binding on HMAs. Multiple peptide sequences in different arrangements (HoMA vs HeMA vs PEGylated HMAs) were also employed to reveal the effect of peptide architecture on their specificity and binding strength. These engineering approaches can be exploited individually or in combination, ultimately allowing precise control over multivalent binding on the engineered surfaces. Given the size of DPCs at ≈10 nm, these hierarchically multivalent architectures would be applicable not only to the glass substrates used in this study, but also to diverse nanoscale (e.g., polymeric nanoparticles, exosomes, liposomes, and various inorganic nanoparticles) and microscale (e.g., microspheres and hydrogels) substrates. Such combinations have great potential to pave the new way in engineering novel platforms for various biomedical applications, including drug delivery, tissue engineering, biosensing, and liquid biopsy.

## 4 Experimental Section

### Materials

Fmoc‐amino acids and coupling reagents were purchased from either Anaspec (Fremont, CA) or Novabiochem (Germany). PEG500 (NH_2_‐(PEG)‐COOH, 0.5 kDa), PEG2000 (2 kDa), and PEG5000 (5 kDa) were purchased from Nektar Therapeutics (Huntsville, AL). G7 PAMAM dendrimers were purchased from Dendritech Inc. (Midland, MI). CellTracker Green and PBS solution were obtained from Thermo Fisher Scientific (Waltham, MA). Fetal bovine serum (FBS) and antibiotic mixture (penicillin/streptomycin) were acquired from Invitrogen. Dulbecco's modification of Eagle's medium (DMEM), DMEM/Ham's F‐12 50/50 mix (DMEM/F‐12), and Roswell Park Memorial Institute (RPMI) medium were purchased from Corning (Manassas, VA). F‐12 nutrient mixture (Ham's F12) and trypsin‐EDTA were obtained from Gibco (Grand Island, NY). Recombinant human EpCAM was purchased from R&D Systems. All other chemicals were obtained from Sigma‐Aldrich (St. Louis, MO), unless otherwise stated.

### Peptide Synthesis

Rink Amide MBHA resin LL or 2‐chlorotrityl chloride resin (Novabiochem, Germany) were used as a scaffold for peptide synthesis. Standard Fmoc chemistry was used for the peptide synthesis, as has been previously reported.^[^ ^32^ ^]^ For the final deprotection and cleavage of the peptide from resin, the resin‐bound peptides were treated with a cleavage cocktail (trifluoroacetic acid (TFA):thioanisole:ethanedithiol (EDT) at a ratio of 95:2.5:2.5, 2 mL) at room temperature for 2 h, which was followed by precipitation with *tert*‐butyl methyl ether. The resulting peptides were purified using reverse‐phase HPLC at room temperature (mobile phase of water/acetonitrile with 0.1% TFA). Matrix‐assisted laser desorption/ionization time‐of‐flight (MALDI‐TOF) mass spectrometry (AXIMA, Shimadzu, Japan) with *α*‐cyano‐4‐hydroxycinnamic acid (CHCA) matrix was used to quantitatively measure molecular weights of the final peptides. The final concentrations of all peptide‐containing solutions were quantified using an ultraviolet–visible (UV–vis) spectrophotometer.

### Slide Preparation

Polydimethylsiloxane (PDMS) gaskets having three discrete wells (Figure S4a, Supporting Information) were utilized to designate the peptide‐functionalized regions on epoxy‐coated glass slides. Each surface was functionalized as follows: 1) glycine/PEG–peptide surfaces were prepared by sequential immobilization of glycine or PEG (100 × 10^−6^ m, overnight) and peptides (300 × 10^−6^ m, overnight) using EDC (3.8 mg mL^−1^)/NHS (4.4 mg mL^−1^) chemistry. 2) HMAs were prepared by immobilizing G7 PAMAM dendrimers (1 mg mL^−1^; 200 µL/2 mm^2^) on epoxide glass slides, followed by carboxylation of amine end groups using an excessive amount of succinic anhydride (1 mg mL^−1^, overnight). Peptides were then immobilized on the surface using EDC/NHS chemistry for 24 h. For some HMAs, the carboxylated dendrimers were PEGylated prior to peptide conjugation using EDC/NHS chemistry. 3) Hetero‐HMAs were prepared by immobilizing peptide‐functionalized dendrimers on epoxy‐coated glass slides. For peptide‐functionalization, G7 PAMAM dendrimers were purified, partially acetylated (60% equivalence for the number of amine groups on the dendrimer surface), fluorescently labeled (Rhodamine, 5 molar equivalent to the dendrimer), and succinylated, following previously published protocols.^[^ ^33^ ^]^ Peptides were conjugated to the dendrimers using EDC/NHS chemistry for 24 h. A 1:1:1 mixture solution of pEP1, pEG1, and pHE2 (finally 300 × 10^−6^ m) was used for the development of HeDPCs. The peptide‐functionalized dendrimers were then conjugated to the epoxy‐coated glass slides via reactions between epoxy groups and hydroxyl groups of the peptides in deionized water (pH 11, overnight). 4) HeMA‐PEG was prepared by immobilizing and PEGylating G7 PAMAM dendrimers on epoxide glass slides, followed by peptide conjugation (pEP1:pEG1:pHE1 = 1:1:1, 300 × 10^−6^ m) using EDC/NHS chemistry.

### Cell Culture

Three different human breast cancer cell lines, MCF‐7, MDA‐MB‐468, and SUM‐52, and a human T lymphocyte cell line Jurkat were used for this study. The cancer cells were grown as a monolayer under humidified condition with 5% CO_2_ at 37 °C. Jurkat cells were grown in suspension under the same humidified condition. Cells were incubated until they reached 50–70% confluence. Cell culture media for each cell line was as follows: 1) MCF‐7 cells were cultured in DMEM media supplemented with 10% FBS (v/v) and 1% penicillin/streptomycin (v/v). 2) MDA‐MB‐468 cells were cultured in DMEM/F12 media supplemented with 10% FBS (v/v) and 1% penicillin/streptomycin (v/v). 3) SUM‐52 cells were cultured in Ham's F12 medium supplemented with 5% FBS (v/v), 1% penicillin/streptomycin (v/v), 0.1% hydrocortisone (v/v), and 0.05% Insulin (v/v). 4) Jurkat cells were cultured in RPMI media supplemented with 10% FBS (v/v) and 1% penicillin/streptomycin (v/v).

### Cell Staining

Cancer cells were gently harvested from a T‐25 flask using trypsin‐EDTA and resuspended in 1 mL DMEM media containing 5 × 10^−6^ m CellTracker green dye, followed by 15 min incubation at 37 °C. The cells were collected using centrifugation at 300 × *g* for 5 min and resuspended in 1 mL fresh DMEM media. For the Jurkat cells, cells were harvested directly from the cell culture media after centrifugation (300 × *g*, 5 min) without using trypsin‐EDTA and stained with CellTracker green dye following the same process.

### Cell Retention Measurement

The functionalized glass slides were assembled into the flow chamber having two discrete channels (Figure S4b,c, Supporting Information). Cells were infused into the flow chamber using a syringe pump (New Era pump 505 Systems Inc., Farmingdale, NY) at a flow rate of 500 µL min^−1^. Cells were imaged using a 5× objective with an inverted microscope (Zeiss Axiocam 503 mon, Carl Zeiss, Germany) and incubated on the flow chamber for 30 min. Cells were then washed backward with complete DMEM media at a flow rate of 50 µL min^−1^ (0.36 dyne cm^2^) for 20 min and the slides were scanned once more. The retention efficiency (*E*_Ret_) was determined as a ratio of cells remaining on the surface after washing.

### AFM Force Spectroscopy

HoMA and HeMA surfaces were prepared as described in the previous section. A single gold‐coated silicon nitride probe was incubated with a mixture of carboxyl‐PEG‐thiol (7500 MW; 0.05 mg mL^−1^) and methoxy‐PEG‐thiol (5000 MW; 5 mg mL^−1^) in deionized water for 12 h. The probes were then functionalized with recombinant human EpCAM (5 µg mL^−1^ in PBS solution) overnight at 4 °C. The spring constant of the probe was 87.14 pN nm^−1^, which was determined by the thermal noise method.

Force mapping and analysis were conducted using an Asylum Infinity Bio system (Oxford Instruments), with both probe and sample submerged in PBS solution. 32 × 32 grid of force curves were obtained in desired area of 10 × 10 µm^2^ for HoMA and HeMA surface, respectively. Force curves consisted of a 2 µm approach at a velocity of 2 µm s^−1^, 1 s dwell, and retraction at a velocity of 2 µm s^−1^.

### CD Analysis

CD spectra were collected using an Aviv model 420 Circular Dichroism spectrometer (Aviv Biomedical, Lakewood, NJ). Samples (20 × 10^−6^ m) dissolved in 400 × 10^−6^ m PBS (pH 7.4) were analyzed from 260 to 200 nm using a 1 mm path length quartz cuvette at room temperature.

### CTC/Cancer Cell Capture

The complete cell capture slides, consisting of capture regions with either PD‐L1‐targeting peptides or antibodies and E‐Selectin‐functionalized cell rolling regions in between the capture regions, were loaded into a flow chamber (Figures S2–S4, Supporting Information). PBMC layers or cell suspensions were withdrawn through the flow channels in a chamber at 0.36 dyne cm^−2^ for 20 min. The captured cells were incubated in a flow chamber for 5 min and washed in a reverse direction at twice the capture flow rate (0.72 dyne cm^−2^) for 20 min. For CTC analysis, capture slides were gently disassembled from the flow chamber and co‐stained with CK (red), CD45 (green), and DAPI (blue), as described in the previous publication.^[^ ^25^ ^]^ For in vitro samples, capture efficiency was determined as the ratio of the cells captured on the surface compared to their initial count, which was ≈2500 cells per test (7500 cells per test when analyzing the spatial distribution of the captured cancer cells).

### AFM Surface Topography

The surface morphology of HoMA and HeMA was scanned using Asylum MFP‐3D Infinity Biosystem (Oxford Instruments, Santa Barbara, CA). The silicon probe (OLYMPUS AC160TS‐R3) with spring constant of ≈26 N m^−1^ was used at a resonant frequency of 300 kHz to measure the mean DPC occupancy. Meanwhile, the probe with spring constant of ≈60 N m^−1^ was used for analyzing the surface roughness and imaging the DPCs. The mean DPC occupancy, surface roughness analysis, and DPC size measurement were all calculated based on the scans obtained from more than three independent square images.

### UV–Vis Absorption Spectroscopy

UV–vis absorption spectra were recorded using Beckman Coulter DU800 UV/Visible spectrophotometer. UV–vis absorption spectra of DPCs were recorded at a step size of 1 nm in the 200–800 nm range.

### XPS

Immobilization of dendrimers, PEG linkers, and peptides were confirmed by analyzing atomic composition of each surface. XPS analysis was conducted using K alpha X‐ray photoelectron spectrometer (Thermo Fishers, Waltham, MA). The spectral features of C 1s, N 1s, O 1s, and Si 2p regions were recorded in the constant analyzer energy (CAE) mode with pass energy of 50.0 eV and step size of 0.20 eV.

### Contact Angle Measurement

Contact angles on modified epoxy glass slides were recorded using a Dataphysics OCA 15 plus Contact Angle measuring device equipped with SCA 20 software (Filderstadt, Germany) based on sessile drop method. A total of five samples were tested for each configuration using 8 µL distilled de‐ionized (DDI) water droplet at the ejection speed of 1 µL s^−1^.

### MD Simulation

MD simulations were performed with the NAMD2.12 package.^[^ ^35^ ^]^ The CHARMM36 force field was used to model peptide atoms and the generalized CHARMM force field was used to model other atoms.^[^ ^36^, ^37^ ^]^ The particle mesh Ewald (PME) method was applied for the assessment of long‐range Coulombic interactions, with a grid space of 1Å.^[^ ^38^ ^]^ Long‐range interactions were evaluated at every^[^ ^35^ ^]^ 1 (van der Waals) and 2 (Coulombic) time steps. All simulations used the NPT ensemble, p = 1 atm, T = 310 K, γLang =0.01 ps‐1, and a time step of 2 fs. The systems were first minimized for 5,000 steps, heated for 2,000 steps, and then equilibrated.

All simulations were done in a physiological solution of [NaCl] = 0.15 M. To mimic the experimental used structures, we constructed replicated the same dendrimers as in experiments. The first dendrimer, which did not have any PEG spacers consisted of 74 peptide ligands. The next dendrimer had PEG spacers 8 links long, yieldin,g a PEG mass 0.5 kDa on each ligand, with a total of 56 ligands. The next dendrimer had PEG spacers 45 links long, yielding a PEG mass of 2 kDa on each ligand, with a total of 81 ligands. The final dendrimer had PEG spacers 110 links long, yielding a PEG mass of 5 kDa on each ligand, with a total of 90 ligands.

### Statistical Analysis

The data obtained from this study were used without preprocessing unless noted otherwise. The bar graph data were presented as mean ± SD. For the box plots, the vertical centerline indicated the median, while the width of the box and error bar represented the IQR and 1.5 times the IQR, respectively. The statistical difference between the obtained data was analyzed using SPSS Statistics 26 (IBM Corp., Armonk, NY). Specifically, a two‐tailed Student's *t*‐test was utilized to assess the difference in cell adhesion properties between the surfaces (*n* ≥ 3). Also, the Shapiro–Wilk normality test was performed to analyze the distribution of large‐sized samples (*n* ≥ 30; i.e., AFM force mapping: *n* = 1024). Significance below 0.05 was determined as asymmetrical. The statistical difference between the samples having an asymmetrical distribution was analyzed using the Mann–Whitney *U* test.

## Conflict of Interest

The authors declare no conflict of interest.

## Supporting information

Supporting Information

Supplemental Video 1

Supplemental Video 2

Supplemental Video 3

Supplemental Video 4

## Data Availability

The data that support the findings of this study are available from the corresponding author upon reasonable request.
