# Supplementary material for: Hierarchically Multivalent Peptide–Nanoparticle Architectures: A Systematic Approach to Engineer Surface Adhesion
Source: Adv Sci (Weinh). 2021 Dec 11;9(4):2103098. doi: 10.1002/advs.202103098 (PMC8811846; doi:10.1002/advs.202103098)
Supplement: Supplementary file 1 — Supporting Information [file ADVS-9-2103098-s002.pdf]

WILEY-VCH

ADVANCED SCIENCE
Open Access

Supporting Information

for *Adv. Sci.*, DOI: 10.1002/advs.202103098

Hierarchically Multivalent Peptide-Nanoparticle Architectures: A systematic approach to engineer surface adhesion

*Woo-jin Jeong, Jiyoon Bu, Roya Jafari, Pavel Rehak, Luke J. Kubiatowicz, Adam J. Drelich, Randall H. Owen, Ashita Nair, Piper A. Rawding, Michael J. Poellmann, Caroline M. Hopkins, Petr Král, and Seungpyo Hong**

Supporting Information

Hierarchically Multivalent Peptide-Nanoparticle Architectures: A systematic approach to engineer surface adhesion

*Woo-jin Jeong, Jiyoon Bu, Roya Jafari, Pavel Rehak, Luke J. Kubiatowicz, Adam J. Drelich, Randall H. Owen, Ashita Nair, Piper A. Rawding, Michael J. Poellmann, Caroline M. Hopkins, Petr Král, and Seungpyo Hong**

Table S1. Statistical significance levels between % retention of surface-bound MCF-7 (top) and Jurkat cells (bottom) on HMA surface with various molecular weight PEG spacers.

	G7-pEP1	G7-$PEG_{0.5k}$-pEP1	G7-PEG_{2k}-pEP1	G7-PEG_{5k}-pEP1
G7-pEP1	-	0.007	0.022	0.939
G7-$PEG_{0.5k}$-pEP1	-	-	0.129	0.003
G7-PEG_{2k}-pEP1	-	-	-	0.009
G7-PEG_{5k}-pEP1	-	-	-	-

	G7-pEP1	G7-$PEG_{0.5k}$-pEP1	G7-PEG_{2k}-pEP1	G7-PEG_{5k}-pEP1
G7-pEP1	-	0.012	0.003	<0.001
G7-$PEG_{0.5k}$-pEP1	-	-	0.455	0.708
G7-PEG_{2k}-pEP1	-	-	-	0.055
G7-PEG_{5k}-pEP1	-	-	-	-

Table S2. Atomic composition, contact angle, and surface roughness of functionalized surfaces measured using XPS, contact angle measurement systems, and AFM, respectively.

	Epoxy	G7-COOH	G7-Pep	G7-$PEG_{0.5k}$	G7-$PEG_{0.5k}$-Pep
Atomic composition (%)					
C 1s	9.66	18.05	29.41	16.94	32.56
N 1s	0.69	3.75	7.12	4.49	7.34
O 1s	63.57	54.68	45.14	55.19	43.01
Si 2p	26.09	23.52	18.32	23.37	17.09
Contact angle (degree)	37.5 ± 1.3	23.0 ± 1.2	52.9 ± 5	21.3 ± 1.4	47.4 ± 4.8
Roughness (rms; pm)	392 ± 27	807 ± 191	1179 ± 68	1043 ± 235	1331 ± 101

pEP1: $_{H}$-KG-**YEVHTYYLD**-$_{OH}$

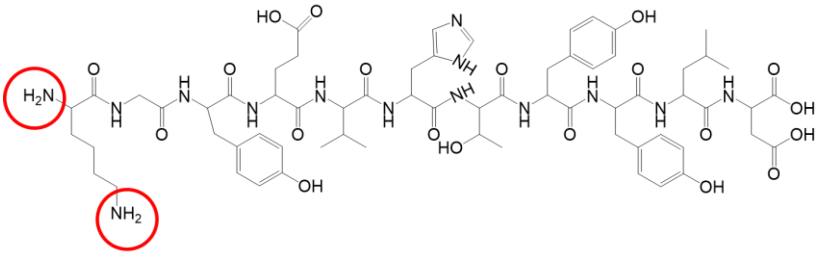

pEG1: $_{Ac}$-**YHWYGYTPQNVI**-GKK-$_{NH2}$

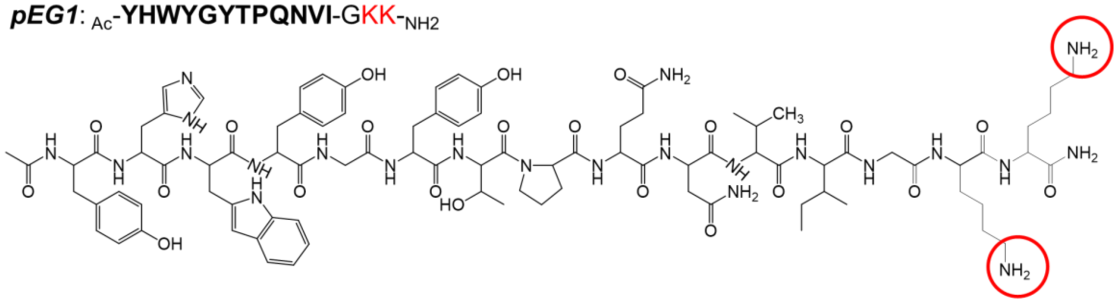

pEG2: $_{H}$-KGSGSG-**LARLLT**-$_{OH}$

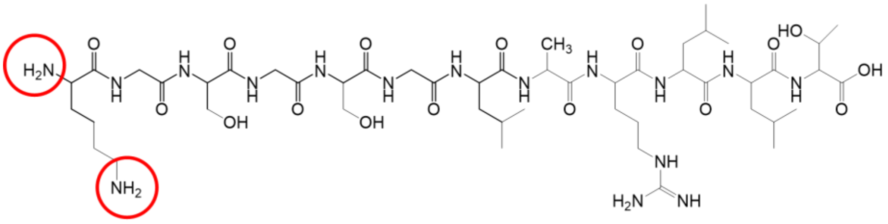

pHE1: $_{Ac}$-**QDVNTAVAW**-GKK-$_{NH2}$

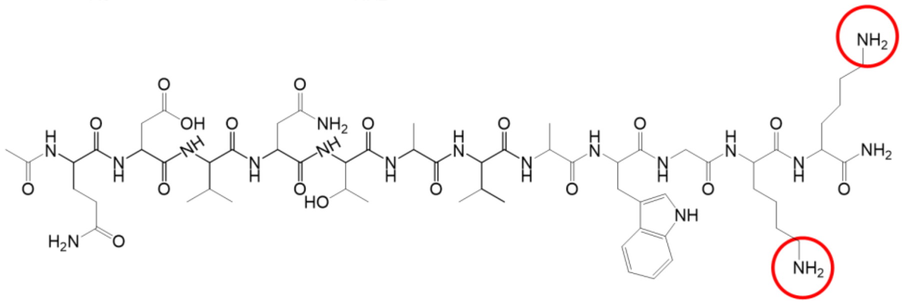

pHE2: $_{H}$-KG-**GQQHYTTP**-$_{OH}$

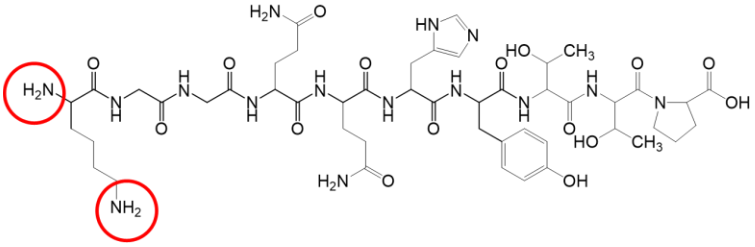

○ = dendrimer conjugation site

Figure S1. Chemical structures of the peptides used in this study. Note that two amine groups were prepared at one terminus of each peptide for effective dendrimer conjugation.

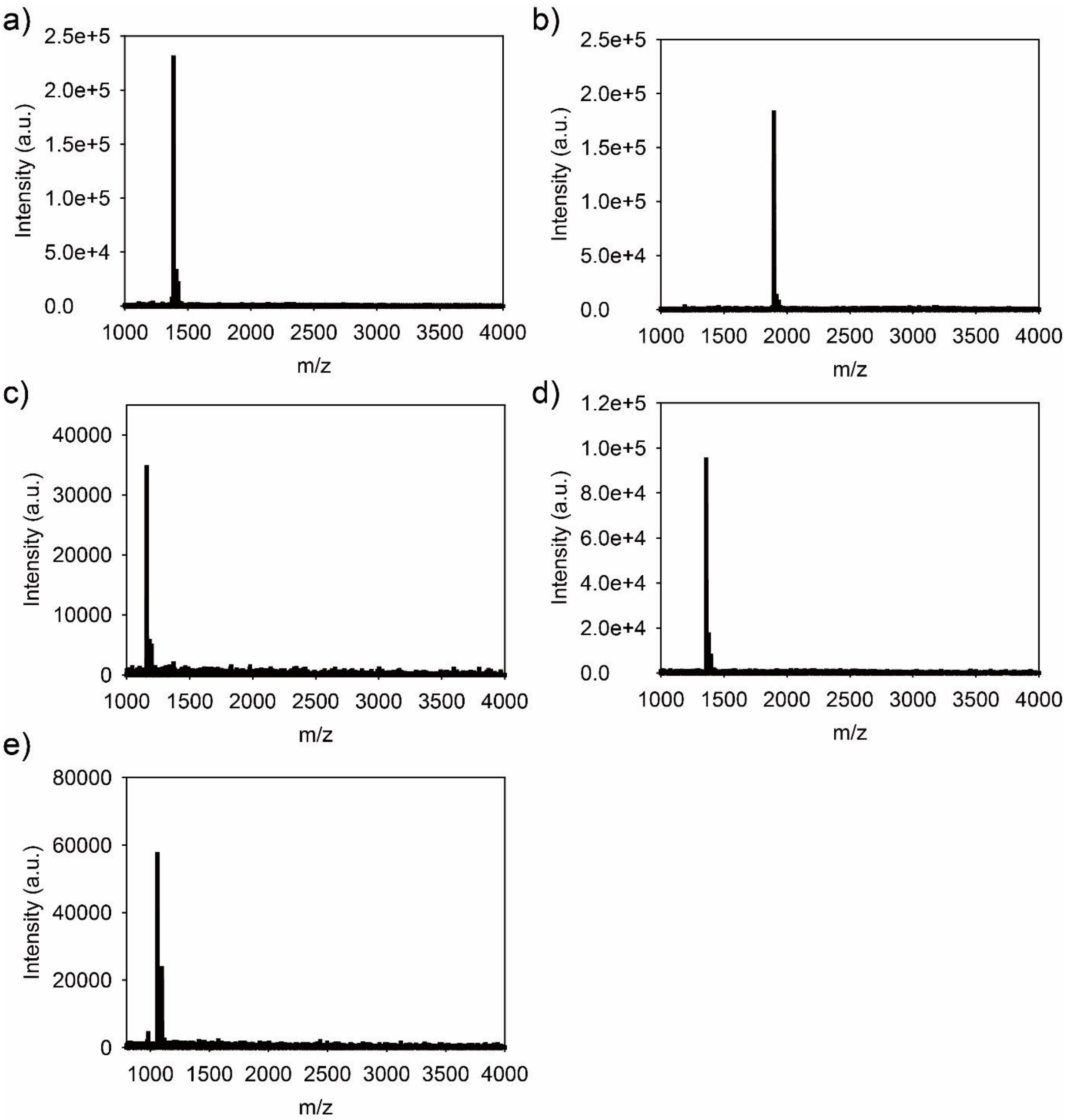

Figure S2. MALDI-TOF MS spectra of (a) pEP1 (calculated MW: 1386.51, observed MW: 1387.40), (b) pEG1 (calculated MW: 1895.12, observed MW: 1896.38), (c) pEG2 (calculated MW: 1158.35, observed MW: 1159.75), (d) pHE1 (calculated MW: 1357.51, observed MW: 1358.78), and (d) pHE2 (calculated MW: 1115.20, observed MW: 1116.67).

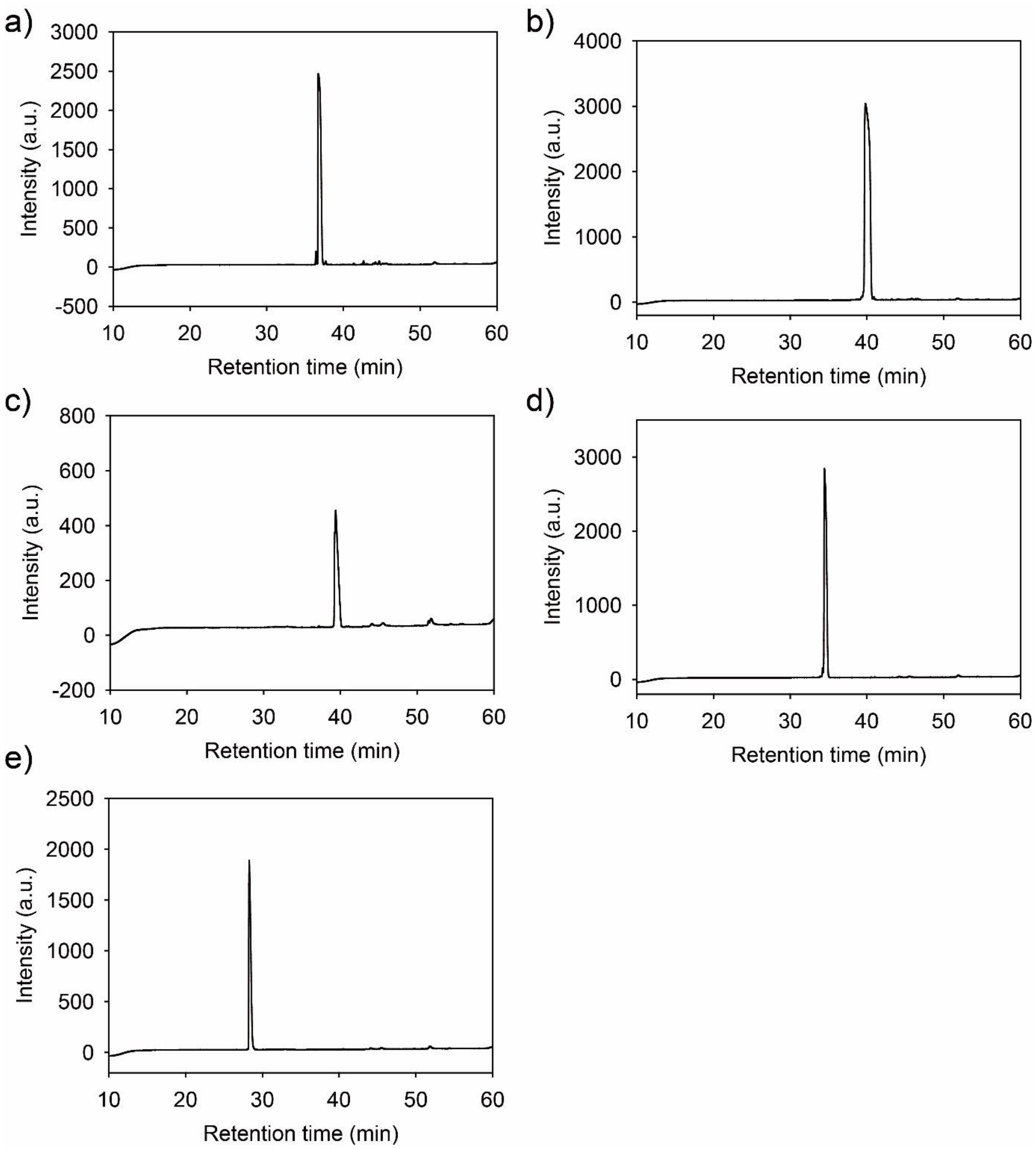

Figure S3. RP-HPLC chromatograms of the purified peptides. (a) pEP1, (b) pEG1, (c) pEG2, (d) pHE1, and (d) pHE2. Condition: C18 column, linear gradient from 0-50% acetonitrile with 0.1% TFA, flow rate of 2 mL/min, and 25 °C.

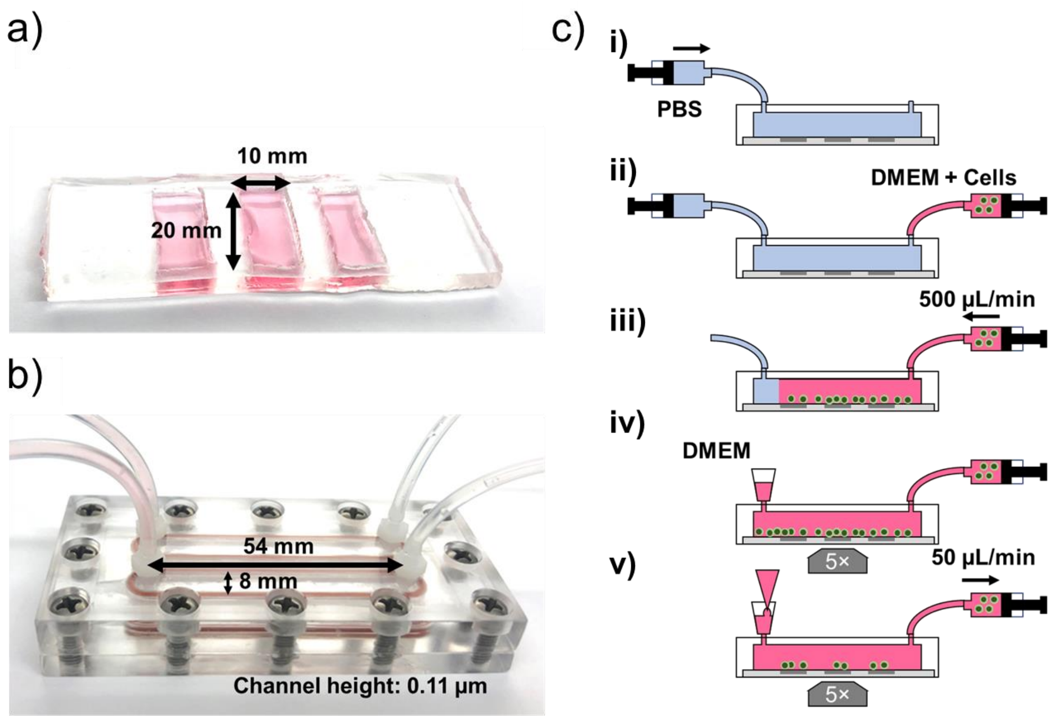

Figure S4. The PDMS gasket and flow chamber used for functionalizing the glass slides and testing the cell retention, respectively: (a) PDMS gaskets having three discrete wells (10 mm × 20 mm) were utilized to designate the peptide-functionalized regions on the epoxide glass slides. (b) Functionalized glass slides were assembled into the flow chamber having two discrete channels. (c) The retention efficiency was measured as followed: i) The flow chamber channels were initially filled with PBS solution by infusing the solution from the port at one end of a channel. ii) The tubing containing cells was connected to the port on the other end. iii) PBS syringe was removed from the tubing and cells were infused through the chamber at a flow rate of 500 µL/min, until the cells reached the outlet port. iv) PBS tubing was removed from the port and media container was connected. The container was filled with fresh DMEM media. The cells were incubated in the chamber for 30 min, while the slides were scanned using 5 × objective. v) The syringe was withdrawn at 50 µL/min for 20 min and re-scanned. The ratio of cells remaining on the surface after washing was determined as a retention efficiency.

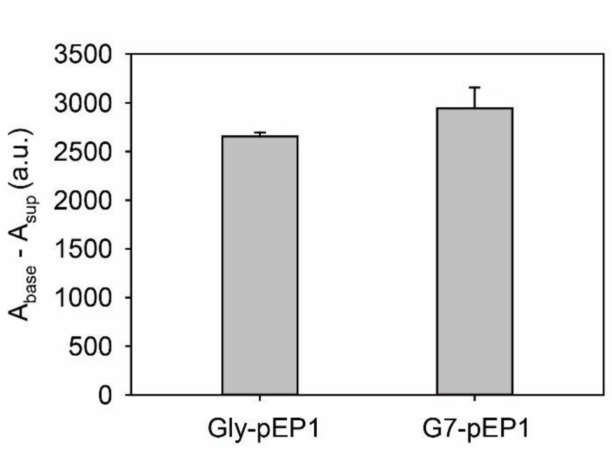

Figure S5. High performance liquid chromatography (HPLC) analysis to measure the quantity of pEP1 conjugated to the Gly-pEP1 and G7-pEP1 surfaces. After peptide conjugation, the supernatant solutions that included free peptides were subjected to the HPLC analysis. Then the peak areas were compared with a free peptide baseline and the difference was used to determine the quantity of conjugated peptides (error bar: standard error, n = 3).

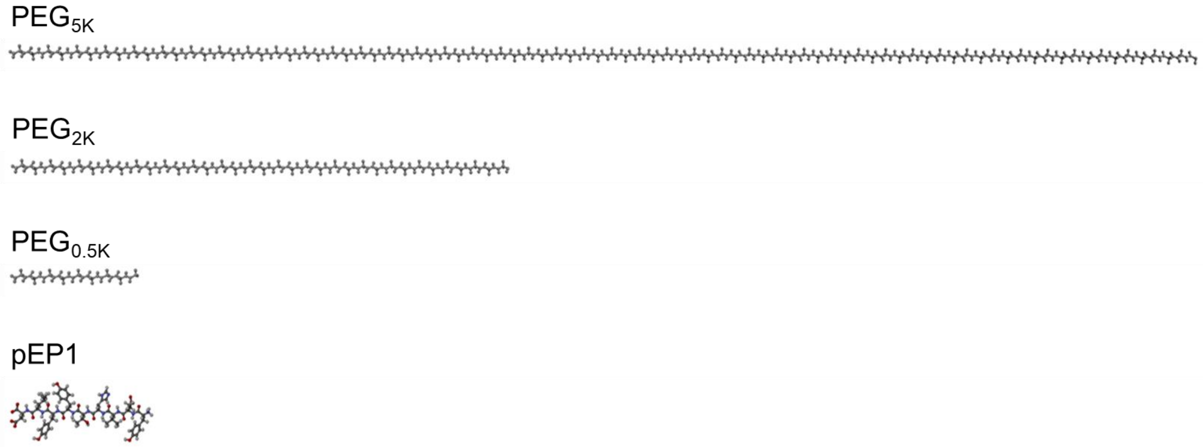

Figure S6. Comparison of the length between PEG_{5K} (31.8 nm), PEG_{2K} (12.7 nm), $PEG_{0.5K}$ (3.1 nm),[1] and pEP1 (3.3 nm).

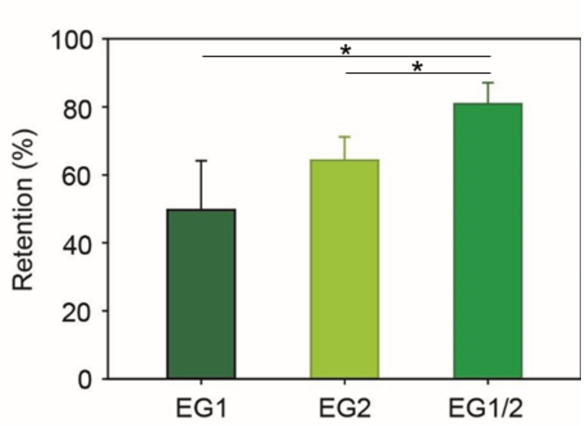

Figure S7. The retention of surface-bound MDA-MB-468 ($EGFR^+$) cells to PEG-pEG1, PEG-pEG2 and PEG-pEG1/2, upon washing at a flow rate of 50 µL/min. Since G7-PEG-pEG1 and G7-PEG-pEG2 already attained significantly high E_{Ret} with the optimized PDC configuration (G7-PEG-peptide) in Figure 2j, the PEG-peptide surfaces were prepared without dendrimer linkers to clarify the advantage of targeting different parts of the EGFR protein.

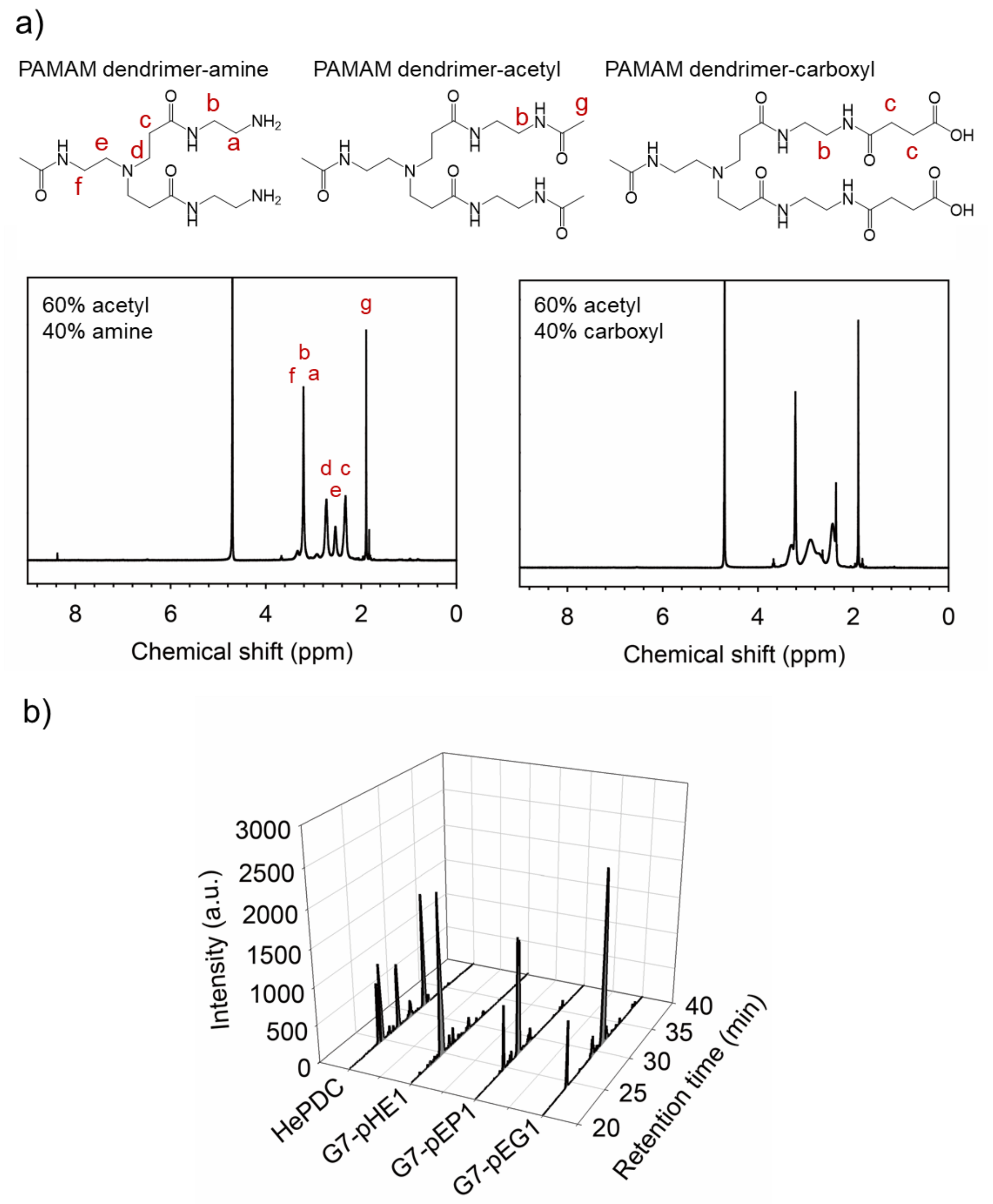

Figure S8. (a) ^{1}H NMR spectra of surface modified G7 PAMAM dendrimers. All measurements were performed in D_2O using a Bruker Advance III HD 400 MHz NMR spectrometer. (b) Determination of peptide number per PDC. After peptide-dendrimer conjugation, unconjugated peptides were separated from the conjugate solution using centrifugal filtration, and the eluants that included free peptides were subjected to HPLC analysis. Then the peak area was compared with a free peptide baseline and the difference was used to determine the number of conjugated peptides. Peptide numbers per DPCs were 74.49 (G7-pEP1), 87.06 (G7-pEG1), 86.28 (G7-pHE1), and 66.69 (HeDPC; G7-pEP1/pEG1/pHE1). HPLC analysis also showed that the ratio of pEP1:pEG1:pHE1 on a single dendrimer was at 1.00:1.11:1.25, which is close to the equal distribution of the three peptides.

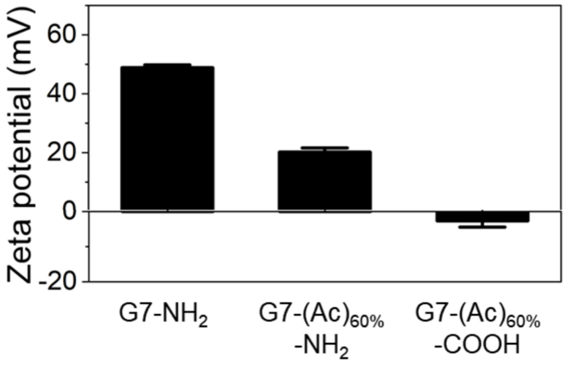

Figure S9. The surface charge density of the dendrimers measured using zeta potential (n = 3).

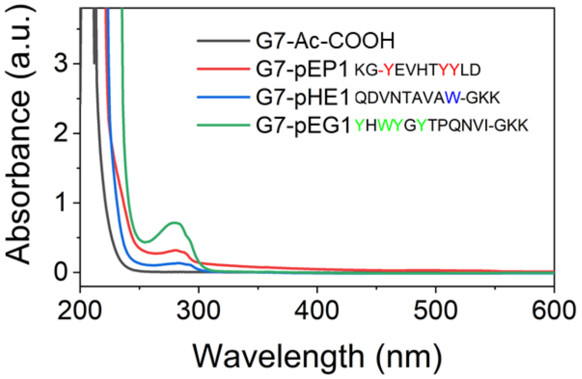

Figure S10. UV-vis absorption spectra of different DPCs. Absorption in the range of 250-300 nm denotes the existence of aromatic side chains (tryptophan, tyrosine, and phenylalanine).

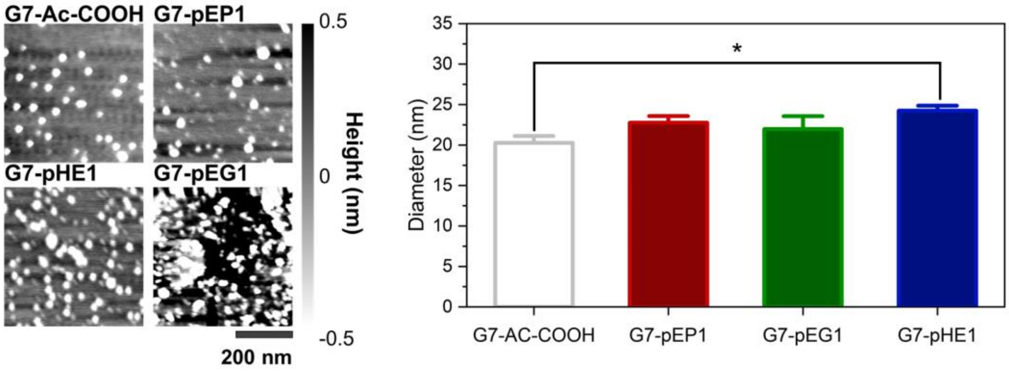

Figure S11. AFM images of surface-adsorbed DPCs obtained in air. The sizes of G7-pEP1 (22.8 nm ± 0.8 nm; p = 0.116), G7-pEG1 (22.0 nm ± 1.6 nm; p = 0.347), and G7-pHE1 (24.3 nm ± 0.6 nm; p =0.019) were all slightly larger than the partially carboxylated G7 dendrimers (20.1 nm ± 0.9 nm). Note that the flattening of the nanoparticles on mica increases the overall sizes of DPCs and dendrimers. The error bars represent the standard error of means obtained from n ≥3 AFM scans.

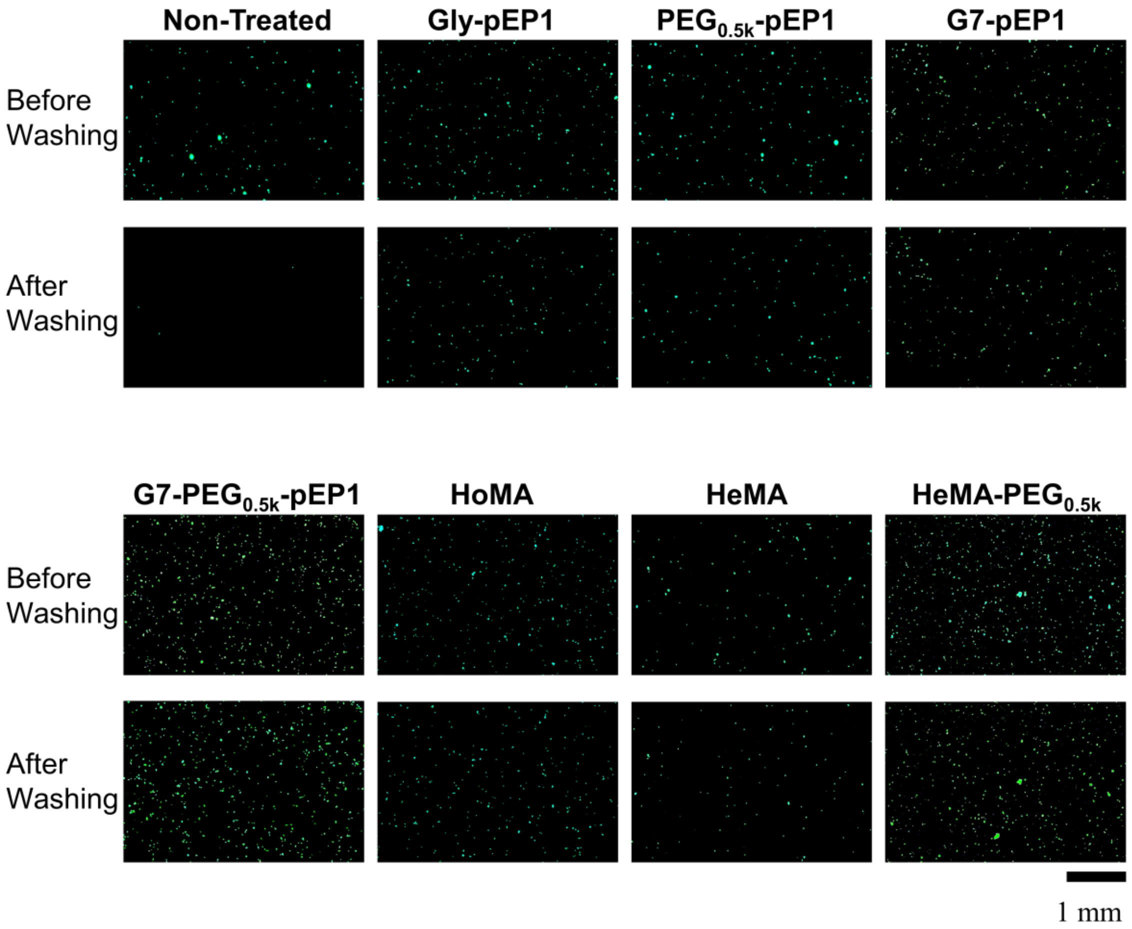

Figure S12. Representative cell retention test images. MCF-7 cells were incubated on different capture surfaces, including non-treated, Gly-pEP1, PEG0.5k-pEP1, G7-pEP1, G7-PEG0.5k-pEP1, HoMA, HeMA, and HeMA-PEG0.5k, followed by washing at a flow rate of 50 µL/min (0.36 dyne/cm^2) for 20 min. Images were obtained before and after washing.

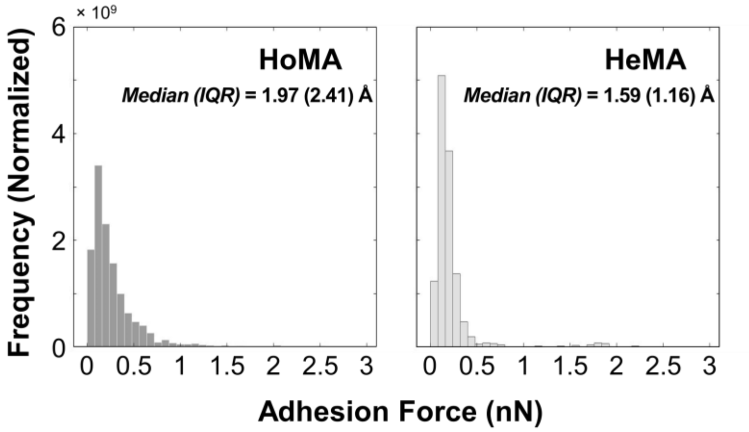

Figure S13. Histogram of the tip-surface adhesion forces. The maximum adhesion forces between the EpCAM-functionalized tip and HMA surfaces (HoMA or HeMA) were collected at a tip pulling velocity of 2 µm/s.

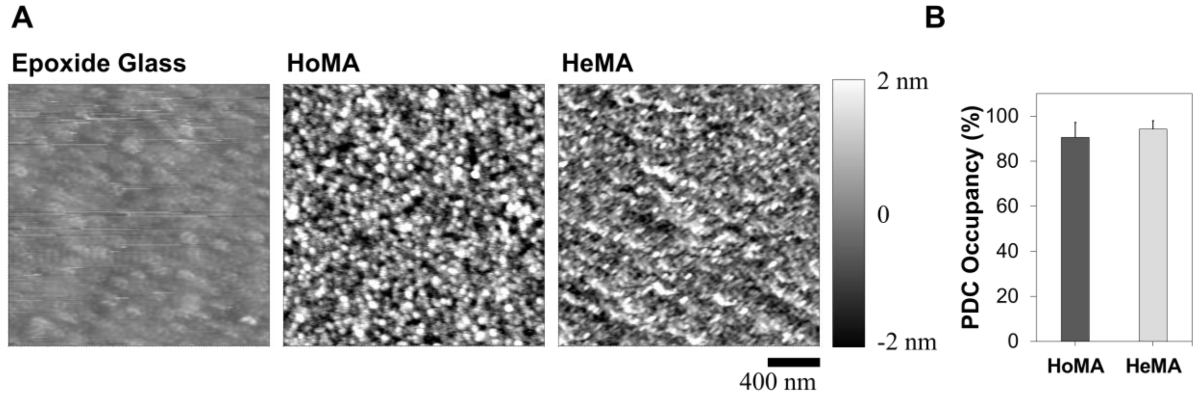

Figure S14. (a) Surface topography of HoMA and HeMA surfaces, mapped using non-contact mode AFM. (b) Coverage of PDCs on HoMA and HeMA surfaces, measured from AFM images ($n \geq 3$).

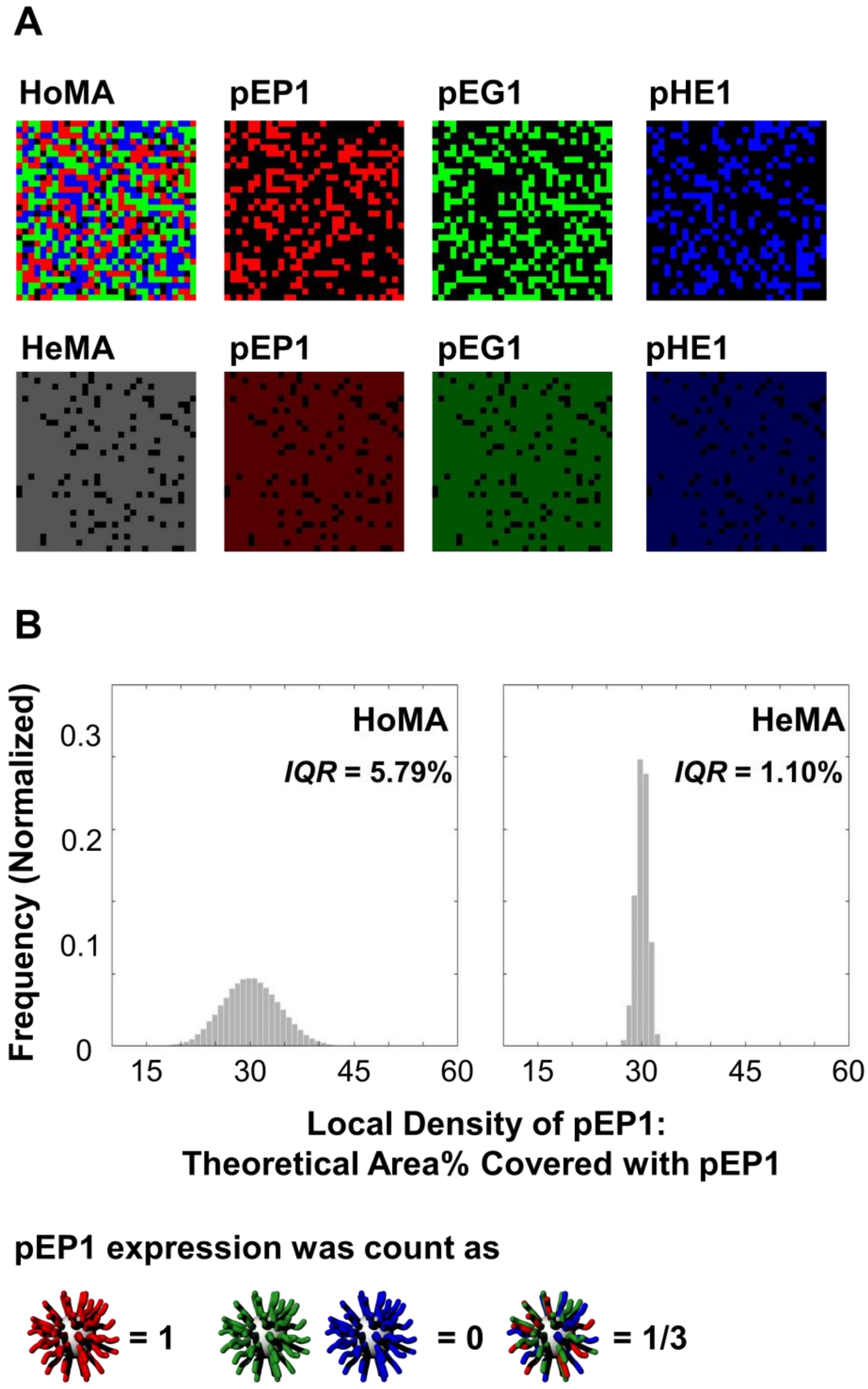

Figure S15. (a) Computer-aided random PDC distributions, representing the HoMA and HeMA structures. The HoMA structure was constructed by filling 90% of 400^2 pixels with either red, blue, or green, each representing G7-pEP1, G7-pEG1, and G7-pHE1, respectively. Meanwhile, the HeMA structure consists of HePDCs (gray; RGB = (0.33,0.33,0.33)) on 90% of its surface. (b) The histograms for local pEP1 density, constructed by measuring the amount of pEP1 expressions on 10^2 pixels among 400^2-pixels HoMA and HeMA structures.

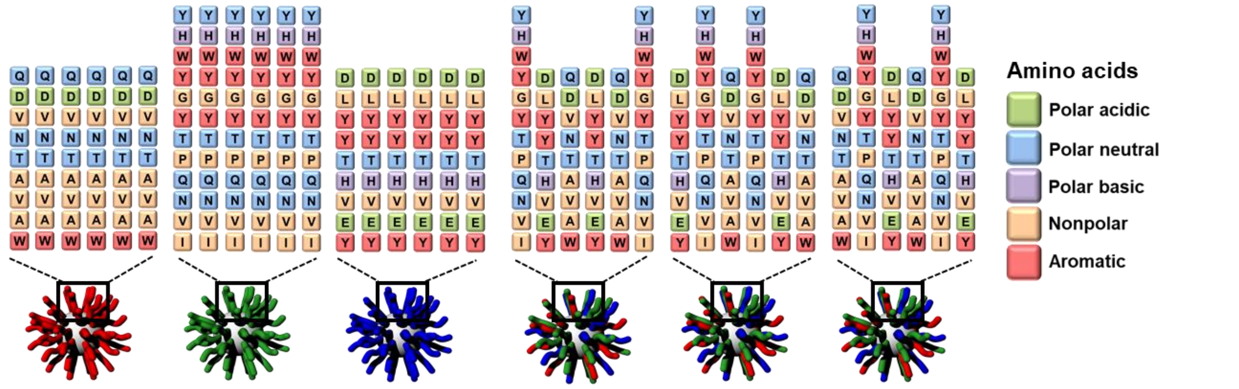

Figure S16. A schematic illustration of different amino acid arrangements on HoMA and HeMA.

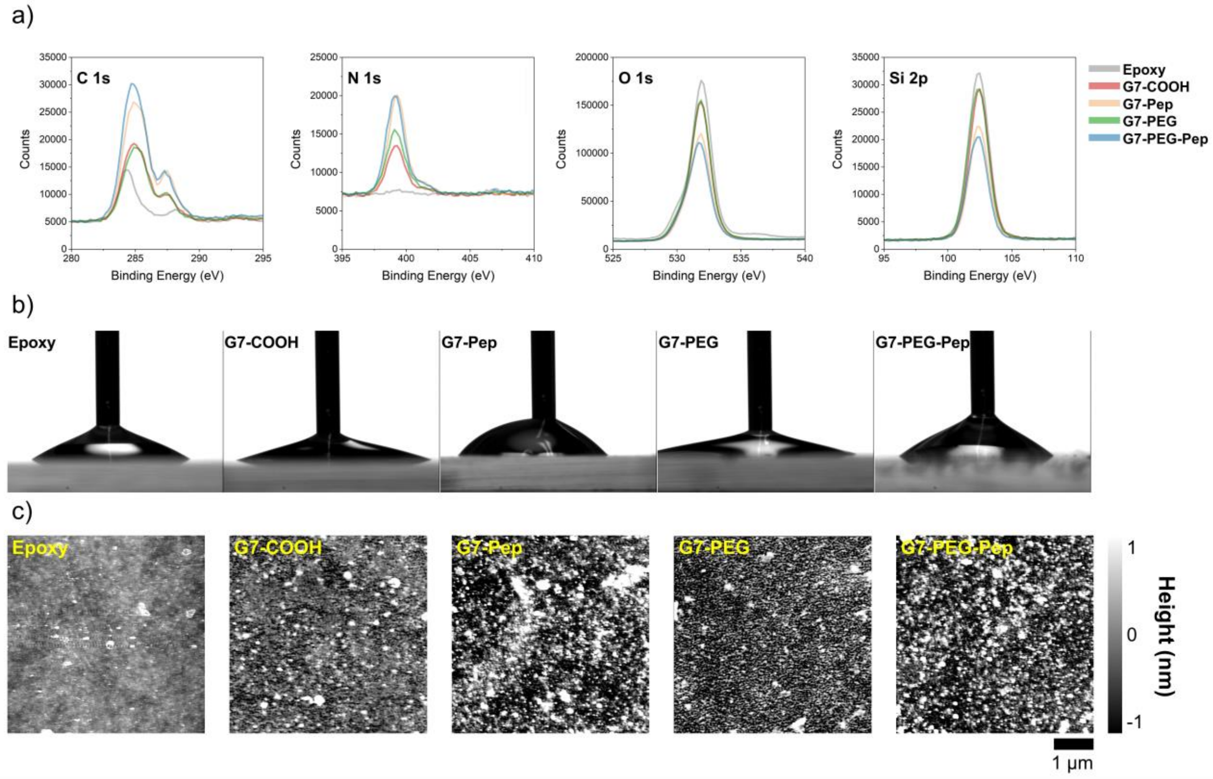

Figure S17. Confirming the surface immobilization of G7 dendrimers, PEG linkers, and peptides on epoxy slide glass. The details are provided in **Table S2**. G7-Pep and G7-PEG-Pep denote HeMA and HeMA-PEG surfaces, respectively.

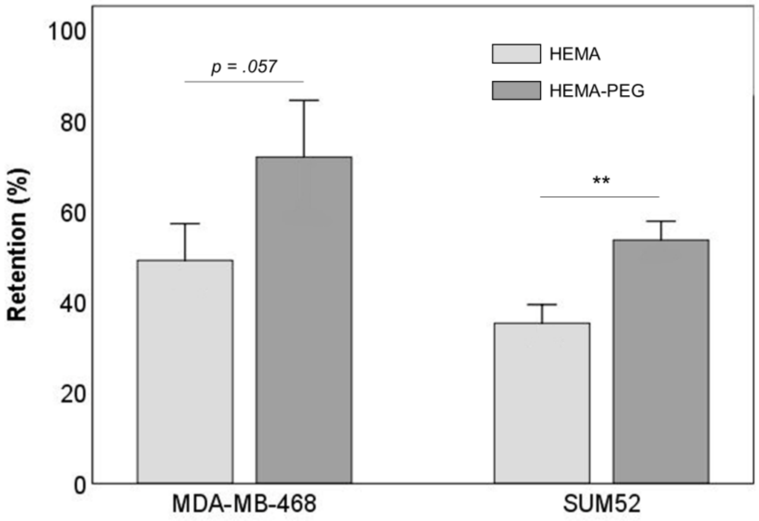

Figure S18. Retention of surface-bound MDA-MB-468 and SUM-52 cells on the HeMA and HeMA-PEG surfaces upon washing.

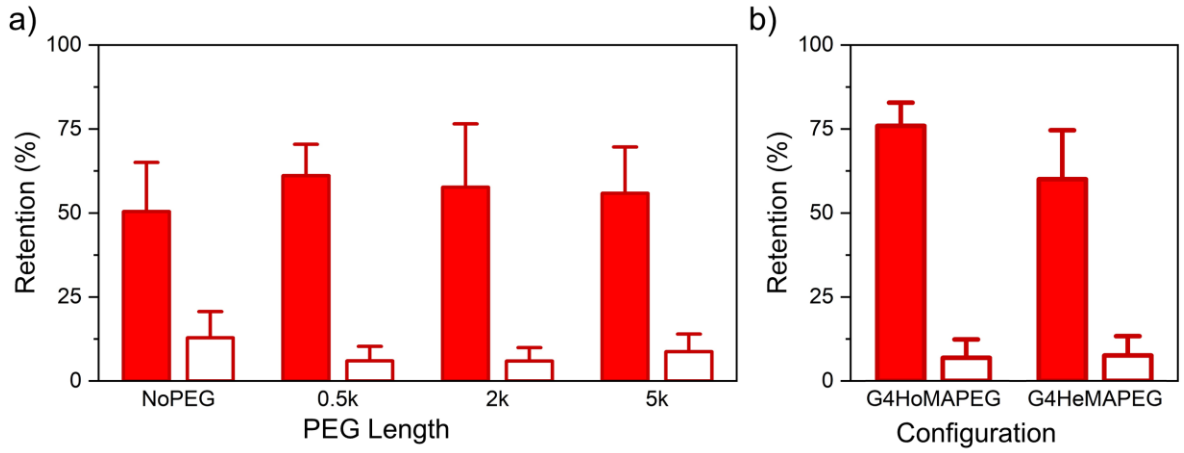

Figure S19. Retention of surface-bound MCF-7 (red) and Jurkat (white) cells on the surface covered with G4-pEP1 conjugates: (a) Depending on the molecular weight of PEG spacer; (b) Depending on the surface arrangement of different peptides (G4-HoMA-PEG vs. G4-HeMA-PEG).

Videos

Video S1. Molecular dynamics simulation of G7-pEP1.
Video S2. Molecular dynamics simulation of G7-$PEG_{0.5k}$-pEP1.
Video S3. Molecular dynamics simulation of G7- PEG_{2k}-pEP1.
Video S4. Molecular dynamics simulation of G7- PEG_{5k}-pEP1.
